# Supplementary material for: Undernutrition combined with dietary mineral oil hastens depuration of stored dioxin and polychlorinated biphenyls in ewes. 1. Kinetics in blood, adipose tissue and faeces
Source: PLoS One. 2020 Mar 31;15(3):e0230629. doi: 10.1371/journal.pone.0230629 (PMC7108735; doi:10.1371/journal.pone.0230629)
Supplement: S1 Table — (DOCX) [file pone.0230629.s001.docx]

| **Table S1. Chemical composition, nutritional values, and dioxin (TCDD) and polychlorinated biphenyls (PCBs) concentrations of feedstuffs.** | | | | |  |
| --- | --- | --- | --- | --- | --- |
| Item | Straw | Hay | Non-contaminated pelleted concentrate | Contaminated concentrate^1^ |  |
| Chemical composition (% DM) | | | | |  |
| Organic matter | 90.0 | 94.5 | 96.5 | 94.2 |  |
| CP | 2.7 | 6.7 | 7.8 | 7.6 |  |
| NDF | 77 | 65 | 29 | 29 |  |
| ADF | 50 | 39 | 13 | 13 |  |
| Fat^2^ |  |  |  |  |  |
| Ether extract | 0.9 | 1.6 | 2.0 | 4.6* |  |
| Lipids | 0.7 | 1.6 | 1.8 | 4.2 |  |
| *In vitro* pepsine-cellulase digestibility (% organic matter) | ND^3^ | ND | 83.1 | ND |  |
| *In vitro* nitrogen degradability 1-h  (% total nitrogen) | ND | ND | 19.0 | ND |  |
| PDIN^4^ | 1.7 | 4.2 | 5.7 | 5.6 |  |
| PDIE^5^ | 4.2 | 5.6 | 10.5 | 10.0 |  |
| Energy (MJ of net energy for lactation.kg dry matter^-1^) | 3.1 | 3.6 | 7.3 | 7.7 |  |
| POPs concentrations (.g DM^-1^) | | | | |  |
| TCDD (pg) | NDetec^6^ | NDetec | NDetec | 67.84* |  |
| PCB 126 (pg) | 0.73 | 0.65 | 0.19 | 67.53* |  |
| PCB 153 (ng) | 0.13 | 0.18 | 0.08 | 67.69* |  |
| ^1^Estimated data considering the level of contaminated rapeseed oil incorporation (2.4%) in 97.6% of non-contaminated pelleted concentrate, except for data with a star (*) which were determined by analysis.  ^2^Ether extract after acid hydrolysis according to the method 920-39 (AOAC, 1997) and lipids: cold extraction according to *Folch et al*., (1957).  ^3^ND: Not determined.  ^4^PDIN: Proteins truly digestible in the small intestine when protein supply is limited.  ^5^PDIE: Proteins truly digestible in the small intestine when energy supply is limited.  ^6^NDetec: Not detected (< Limit of detection). | | | | |  |
