# Supplementary material for: Undernutrition combined with dietary mineral oil hastens depuration of stored dioxin and polychlorinated biphenyls in ewes. 1. Kinetics in blood, adipose tissue and faeces
Source: PLoS One. 2020 Mar 31;15(3):e0230629. doi: 10.1371/journal.pone.0230629 (PMC7108735; doi:10.1371/journal.pone.0230629)
Supplement: S2 Table — (DOCX) [file pone.0230629.s002.docx]

| **Table S2. Dioxin (TCDD) and polychlorinated biphenyls (PCBs) concentrations and burdens in blood serum and pericaudal subcutaneous adipose tissue of exposed ewes^1^** | | | | | | | | | | | | | | | | | |
| --- | --- | --- | --- | --- | --- | --- | --- | --- | --- | --- | --- | --- | --- | --- | --- | --- | --- |
| Item | Treatment | Buffering period (mean±SD) | Depuration period | | | | | | | | | | | | | |  |
|  |  |  | Day | | | | | | | | | SEM | | *P*-value | | | |
|  |  |  | 7 | | 21 | | 35 | | 57 | |  | | T^2^ | | Day | T^2^×Day |  |
| Serum | | | | | | | | | | | | | | | | | |
| Total lipids (mg.dL^-1^) | CTL | 238±26 | 218 |  | 209 |  | 238 |  | 229 |  | 12 | | 0.19 | | 0.29 | 0.64 |  |
|  | UFMO | 193±25 | 175 |  | 199 |  | 197 |  | 209 |  |  |  |  |  |  |  |  |
| POPs concentrations (.g lipid^-1^) | | | | | | | | | | | | | | | | | |
| TCDD (pg) | CTL | 10.6±3.4 | 8.5 |  | 7.9 |  | 6.9 |  | 7.2 |  | 0.9 | | 0.67 | | 0.49 | 0.88 |  |
|  | UFMO | 10.3±1.5 | 8.8 |  | 7.8 |  | 7.7 |  | 8.4 |  |  |  |  |  |  |  |  |
| PCB 126 (pg) | CTL | 22.9±5.3 | 21.3^a^ |  | 16.1^ab^ | * | 19.0^ab^ |  | 14.9^b^ |  | 0.8 | | 0.05 | | 0.06 | 0.50 |  |
|  | UFMO | 27.2±7.6 | 21.9 |  | 22.2 |  | 19.8 |  | 17.8 |  |  |  |  |  |  |  |  |
| PCB 153 (ng) | CTL | 9.7±3.0 | 7.7 |  | 6.3 |  | 5.8 |  | 6.6 |  | 0.6 | | 0.84 | | 0.08 | 0.51 |  |
|  | UFMO | 10.0±0.7 | 6.9 |  | 5.9 |  | 6.4 |  | 7.9 |  |  |  |  |  |  |  |  |
| Pericaudal subcutaneous adipose tissue | | | | | | | | | | | | | | | | | |
| POPs concentrations (.g lipid^-1^) | | | | | | | | | | | | | | | | | |
| TCDD (pg) | CTL | 10.7±3.3 | 11.1^c^ |  | 15.0^b^ |  | 16.1^b^ | † | 20.5^a^ | * | 1.6 | | 0.07 | | <0.001 | 0.05 |  |
|  | UFMO | 9.5±3.8 | 12.9^d^ |  | 16.7^c^ |  | 21.8^b^ |  | 31.5^a^ |  |  |  |  |  |  |  |  |
| PCB 126 (pg) | CTL | 14.8±3.7 | 15.3 |  | 17.2 |  | 18.2 |  | 19.4 | * | 1.8 | | 0.10 | | <0.01 | 0.12 |  |
|  | UFMO | 14.4±4.8 | 16.5^c^ |  | 18.8^c^ |  | 24.1^b^ |  | 31.2^a^ |  |  |  |  |  |  |  |  |
| PCB 153 (ng) | CTL | 9.3±2.3 | 10.5^b^ |  | 12.9^ab^ |  | 15.6^ab^ |  | 22.2^a^ | * | 2.0 | | 0.18 | | <0.001 | 0.40 |  |
|  | UFMO | 9.5±3.1 | 10.6^c^ |  | 13.9^bc^ |  | 20.5^b^ |  | 33.7^a^ |  |  |  |  |  |  |  |  |
| Estimated lipid weight (g) | CTL | 94±38 | 91^ab^ |  | 93^a^ | † | 79^b^ | * | 83^ab^ | ** | 8 | | 0.04 | | <0.001 | <0.001 |  |
|  | UFMO | 110±45 | 89^a^ |  | 68^b^ |  | 48^c^ |  | 33^d^ |  |  |  |  |  |  |  |  |
| POPs burdens | | | | | | | | | | | | | | | | | |
| TCDD (ng) | CTL | 1.10±0.68 | 1.04^c^ |  | 1.40^ab^ |  | 1.22^bc^ |  | 1.65^a^ | ** | 0.11 | | 0.10 | | 0.05 | 0.02 |  |
|  | UFMO | 0.95±0.36 | 1.06 |  | 1.08 |  | 0.97 |  | 1.02 |  |  |  |  |  |  |  |  |
| PCB 126 (µg) | CTL | 1.49±0.85 | 1.44 |  | 1.63 | † | 1.43 |  | 1.60 | * | 0.13 | | 0.10 | | 0.05 | <0.01 |  |
|  | UFMO | 1.53±0.67 | 1.43^a^ |  | 1.24^ab^ |  | 1.07^bc^ |  | 0.97^c^ |  |  |  |  |  |  |  |  |
| PCB 153 (µg) | CTL | 0.94±0.53 | 0.96^b^ |  | 1.16^b^ |  | 1.17^b^ |  | 1.81^a^ | * | 0.12 | | 0.07 | | <0.01 | 0.10 |  |
|  | UFMO | 1.00±0.45 | 0.92 |  | 0.89 |  | 0.84 |  | 1.10 |  |  |  |  |  |  |  |  |
| ^1^Four ewes received a control well-fed and non-supplemented treatment (CTL), while five ewes received an underfed and mineral oil supplemented treatment (UFMO).  ^2^T: Treatment.  ^a-d^Means within a row and between days with different letters differ at *P*≤0.05.  †,*Means within a column and between treatments tend to differ at *P* ≤ 0.10 (†) or differ at *P* ≤ 0.05 (*). | | | | | | | | | | | | | | | | |  |
