# Supplementary material for: Undernutrition combined with dietary mineral oil hastens depuration of stored dioxin and polychlorinated biphenyls in ewes. 1. Kinetics in blood, adipose tissue and faeces
Source: PLoS One. 2020 Mar 31;15(3):e0230629. doi: 10.1371/journal.pone.0230629 (PMC7108735; doi:10.1371/journal.pone.0230629)
Supplement: S3 Table — (DOCX) [file pone.0230629.s003.docx]

**Table S3. Faecal concentrations and daily flows of lipids and POPs (in dry matter and lipids basis) of exposed ewes^1^**

| Item^2^ | Treatment | Buffering period (Day -1) | Depuration period (Day) | | | |
| --- | --- | --- | --- | --- | --- | --- |
|  |  |  | 7 | 21 | 35 | 55 |
| Lipid concentrations (.g DM^-1^) | CTL | 2.1 | 2.1 | 2.2 | 2.1 | 2.4 |
|  | UFMO | 2.1 | 12.4 | 13.9 | 14.4 | 13.1 |
| POPs DM-normalized concentrations (.g DM^-1^) | | | | | | |
| TCDD (pg) | CTL | 0.48 | 0.45 | 0.32 | 0.43 | 0.33 |
|  | UFMO | 0.49 | 0.72 | 0.75 | 0.84 | 0.96 |
| PCB 126 (pg) | CTL | 0.59 | 0.58 | 0.58 | 0.60 | 1.49 |
|  | UFMO | 0.71 | 1.12 | 1.23 | 1.49 | 1.45 |
| PCB 153 (ng) | CTL | 0.41 | 0.42 | 0.41 | 0.35 | 1.06 |
|  | UFMO | 0.52 | 0.75 | 0.85 | 0.99 | 1.34 |
| POPs lipid-normalized concentrations (.g lipid^-1^) | | | | | | |
| TCDD (pg) | CTL | 22.4 | 21.5 | 14.2 | 20.5 | 13.6 |
|  | UFMO | 23.8 | 5.8 | 5.4 | 5.8 | 7.3 |
| PCB 126 (pg) | CTL | 27.7 | 28.2 | 26.1 | 28.6 | 61.6 |
|  | UFMO | 34.7 | 9.1 | 8.8 | 10.4 | 11.1 |
| PCB 153 (ng) | CTL | 17.8 | 19 | 17 | 15.5 | 40.8 |
|  | UFMO | 23.5 | 5.7 | 5.7 | 6.4 | 9.6 |

^1^See footnote 1 of Table S2.

^2^Faecal concentrations data are individual results obtained from pools of faeces by treatment and by date.
