# Supplementary material for: Undernutrition combined with dietary mineral oil hastens depuration of stored dioxin and polychlorinated biphenyls in ewes. 1. Kinetics in blood, adipose tissue and faeces
Source: PLoS One. 2020 Mar 31;15(3):e0230629. doi: 10.1371/journal.pone.0230629 (PMC7108735; doi:10.1371/journal.pone.0230629)
Supplement: S1 File — (DOCX) [file pone.0230629.s004.docx]

**S1 File. Contaminated rapeseed oil and mineral oil description.**

The contaminated rapeseed oil was prepared by mixing thoroughly 7.0 μg of TCCD (10 μg/mL toluene, Sigma-Aldrich, Bellefonte, PA, USA), 7.0 μg of PCB 126 (100 μg/mL iso-octane, LGC Standards, Molsheim, France) and 7.0 mg of PCB 153 (powder of purity >99%, LGC Standards) in 2.5 kg of commercial food-grade rapeseed oil. Uncontaminated and contaminated concentrates were in the form of pellets of 2.5-mm diameter produced by the mill of the “Pôle d’Expérimentation Avicole de Tours” (INRA UE 1295, France), as described by Lerch et al. (2016). The mineral oil saturated hydrocarbons (i.e. liquid paraffin, Codex 68 grade, kinematic viscosity at 40°C: 60-79 mm²/s, IGOL, Amiens, France) was a mix of linear and branched alkanes (number of carbons >25) with very limited digestive absorption (virtually totally non-absorbable lipids).

Lerch S, Guidou C, Thome JP, Jurjanz S. 2016. Non-dioxin-like polychlorinated biphenyls (PCBs) and chlordecone release from adipose tissue to blood in response to body fat mobilization in ewe (Ovis aries). J Agric Food Chem. 64:1212-1220.
