# Supplementary material for: Undernutrition combined with dietary mineral oil hastens depuration of stored dioxin and polychlorinated biphenyls in ewes. 1. Kinetics in blood, adipose tissue and faeces
Source: PLoS One. 2020 Mar 31;15(3):e0230629. doi: 10.1371/journal.pone.0230629 (PMC7108735; doi:10.1371/journal.pone.0230629)
Supplement: S2 File — (DOCX) [file pone.0230629.s005.docx]

**S2 File. POPs analyses method.**

The concentrations of TCDD, PCBs 126 and 153 in feedstuffs, serum, pericaudal subcutaneous adipose tissue (PSAT) and faeces were determined according to ISO/IEC 17025:2005 fully accredited methods (except for faeces), which have been slightly adapted from previously described methods (Antignac et al. 2006; Cariou et al. 2010).

Lipids were extracted from lyophilized feedstuffs (3‑10 g), PSAT (0.3‑0.5 g) and faeces (2.5 g) by pressurized liquid extraction over three successive static cycles (100 bar, 120 °C) (SpeedExtractor, Büchi, Switzerland) with a toluene/acetone 70:30 mixture (*v/v*) (Sigma-Aldrich, Saint-Louis, MO), and determined gravimetrically. Serum (10 mL) was digested with one volume of formic acid at room temperature during 20 min, diluted with one volume of ultrapure water, loaded on a C18 SPE cartridge, washed with 20 mL ultrapure water, dried under vacuum during 2 h and eluted with 20 mL *n*‑hexane.

All extracts were purified and fractionated using a fully automatic preparation system involving three heated columns: a silver nitrate and sulphuric acid silica column, a carbon column and an alumina column (GO-4HT, Miura, Seoul, Korea). Two fractions were collected, the first one containing TCDD and PCB 126, the second one PCB 153.

Separation and detection of target compounds were achieved by gas chromatography (7890A, Agilent, Santa Clara, CA) coupled to high-resolution mass spectrometry on an electromagnetic sector instrument operating at 10,000 resolution for TCDD and PCB 126 (JMS 800D, Jeol, Tokyo, Japan) or coupled to low-resolution mass spectrometry on a quadrupole instrument for PCB 153 (5975C, Agilent). Instruments were operating in the positive electron impact ionisation mode and the single ion monitoring acquisition mode. Quantification was performed according to the isotopic dilution method (^13^C_12_-labeled internal standards corresponding to each targeted congener; Wellington Laboratories, Guelph, Canada and Cambridge Isotope Laboratories, Tewksbury, MA). No signal interferences due to mineral oil (MO) were observed in any extract, even for faeces.

Limit of quantification (LOQ) were 0.008-0.150 and 0.032-0.6 pg/g dry matter (DM) for feedstuffs, 0.2-0.3 and 0.6-1.1 pg/g lipids for PSAT, 0.030 and 0.120 pg/g DM for faeces, 0.0075 and 0.030 pg/mL for serum, for TCDD and PCB 126 respectively. For all matrices, the concentrations were higher than the LOQ except for the TCDD in forages and non-contaminated pelleted concentrate.

Antignac JP, Marchand P, Gade C, Matayron G, Qannari EM, Le Bizec B, Andre F. 2006. Studying variations in the PCDD/PCDF profile across various food products using multivariate statistical analysis. Anal Bioanal Chem. 384:271-279.

Cariou R, Marchand P, Vénisseau A, Brosseaud A, Bertrand D, Qannari E, Antignac JP, Le Bizec B. 2010. Prediction of the PCDD/F and dl-PCB 2005-WHO-TEQ content based on the contribution of six congeners: Toward a new screening approach for fish samples? Environ Pollut. 158:941-947.
