# Supplementary material for: Undernutrition combined with dietary mineral oil hastens depuration of stored dioxin and polychlorinated biphenyls in ewes. 1. Kinetics in blood, adipose tissue and faeces
Source: PLoS One. 2020 Mar 31;15(3):e0230629. doi: 10.1371/journal.pone.0230629 (PMC7108735; doi:10.1371/journal.pone.0230629)
Supplement: S3 File — (DOCX) [file pone.0230629.s006.docx]

**S3 File. Codes for statistical analyses.**

Data were analysed by ANOVA using the MIXED procedure of SAS 9.3.

- Code for feedstuff/nutrient intakes and energy/protein balances (example of dry matter intake: DMI):

proc mixed ratio covtest ;

class ewe treatment day;

model DMI = treatment day treatment*day  covariate_DMI / DDFM=KenwardRoger outp=residy residual ;

repeated day  /          type= ar(1) sub = ewe ;

random ewe ;

lsmeans treatment / pdiff ;

lsmeans day / pdiff adjust = Tukey ;

lsmeans tratment*day / pdiff adjust = Tukey slice=day ;

run;

- Code for the other parameters (example of TCDD concentration in adipose tissue (AT): [TCDD]AT)

proc mixed ratio covtest ;

class ewe treatment day;

model [TCDD]AT = treatment day treatment*day covariate_[TCDD]AT / DDFM=KenwardRoger outp=residy residual ;

repeated day /          type=sp(pow) (day) sub = ewe ;

random ewe ;

lsmeans treatment / pdiff ;

lsmeans day / pdiff adjust = Tukey ;

lsmeans treatment*day / pdiff adjust = Tukey slice=day ;

 run;
