# Supplementary material for: Undernutrition combined with dietary mineral oil hastens depuration of stored dioxin and polychlorinated biphenyls in ewes. 1. Kinetics in blood, adipose tissue and faeces
Source: PLoS One. 2020 Mar 31;15(3):e0230629. doi: 10.1371/journal.pone.0230629 (PMC7108735; doi:10.1371/journal.pone.0230629)
Supplement: Attachment — Submitted filename: Response to Reviewers.docx [file pone.0230629.s007.docx]

**Subject: Manuscript PONE-D-19-30221: “Undernutrition combined with dietary mineral oil hastens depuration of stored dioxin and polychlorinated biphenyls in ewes. 1. Kinetics in blood, adipose tissue and faeces“**

Dear Editor,

We appreciate all of the constructive comments and thoughtful suggestions provided by the referee. All suggestions arising from the peer-review have been carefully considered during revision of the original manuscript. In all cases, the suggested changes have been carefully considered and implemented in full.

The associate changes are highlighted in red thorough the manuscript labeled 'Revised Manuscript with Track Changes’. A response to the comment from the referee is outlined in the accompanying response indicated by sentences starting with AU:. The line number reported in the response are the one of the 'Revised Manuscript with Track Changes’.

We hope that this revision would allow the manuscript to be considered acceptable for publication.

With our best regards,

L. Rey-Cadilhac and S. Lerch,

On behalf of all co-authors

**Journal Requirements:**

1. Please ensure that your manuscript meets PLOS ONE's style requirements, including those for file naming. The PLOS ONE style templates can be found at <http://www.plosone.org/attachments/PLOSOne_formatting_sample_main_body.pdf> and <http://www.plosone.org/attachments/PLOSOne_formatting_sample_title_authors_affiliations.pdf>

AU: Our initial figures files do not meet the PLOS ONE requirements. Files were reshaped in order to meet the requirements before resubmission of this R1 version. Now, they appear and are readable in the pdf version of the manuscript at submission.

2. The link provided In your Data Availability Statement: <https://doi.org/10.15454/Z6UML7>,  states that the DOI is not found.

AU: The <https://doi.org/10.15454/Z6UML7> is the final link where the data would be available once the paper would be published (access would be opened). Until then, a private link, **where the data can be found until they get public;** is: <https://data.inra.fr/privateurl.xhtml?token=9d8c85ad-263c-4cab-b36d-66d1a0a5c32e>

PLOS defines a study's minimal data set as the underlying data used to reach the conclusions drawn in the manuscript and any additional data required to replicate the reported study findings in their entirety. All PLOS journals require that the minimal data set be made fully available. For more information about our data policy, please see <http://journals.plos.org/plosone/s/data-availability>.

Upon re-submitting your revised manuscript, please upload your study’s minimal underlying data set as either Supporting Information files or to a stable, public repository and include the relevant URLs, DOIs, or accession numbers within your revised cover letter. For a list of acceptable repositories, please see <http://journals.plos.org/plosone/s/data-availability#loc-recommended-repositories>. Any potentially identifying patient information must be fully anonymized.

AU: all the individual experimental data will be made publicly available on the data.inra repository, using the DOI 10.15454/Z6UML7 (available when published).

**Reviewers Comments to the Author**

Reviewer #1: here.

Line 79: Here the author give the three molecules investigated in this study. However, the description above has nothing to do with these molecules. Please rewrite the introduction to focus on the dioxin and PCBs.

AU: A sentence was added in the introduction explaining that PCBs, dioxins, and organochlorine pesticides (hexachlorobenzene and mirex) are all part of the Persistent Organic Pollutants (POPs) list defined in the Stockholm convention. See lines 47-49 and 82-85. They have close physico-chemical (especially their high lipophilicity), and toxicokinetics properties, so that the comparison can be made across POPs molecules regarding the effect of mineral supplementation or undernutrition on toxicokinetic in the introduction section. Moreover, terminology was reviewed thorough the text, retaining only “persistent organic pollutants” as a generic term in order to describe this group of molecules, and no more “lipophilic contaminants” or “lipophilic compounds”. See lines 51 and 91 in the introduction and throughout the main text.

Line 192: Because the concentrations of TCDD and PCBs were very low in the samples, it was difficult to analyze them in small amount of samples. How much volume of blood and serum and other matrix were collected and analyzed in this study? What was the detection limit? Please provide the details.

AU: This information was added to the *S2 File. POPs analysis method.* which described the steps, material and methods for the POPs analysis. For all matrixes, the measured concentrations were higher than the LOD/LOQ except for the TCDD in forages and non-contaminated pelleted concentrate for which the concentrations were under the LOD/LOQ.

Figure 1-4: These figures can’t be displayed in PDF version.

AU: Figures have been reformatted using the Preflight Analysis and Conversion Engine (PACE) digital diagnostic tool as advised. They are now available in the PDF version of the new manuscript.

Please show more data in the manuscript.

AU: Now the Figures 1 to 4 are readable from the main pdf manuscript. Moreover, in order to show more data in the main text, and better balance the data provided between main text and supporting information, the Table S2 about *Ingredients, nutrients and POPs intakes of ewes during the buffering and the depuration periods* was displaced from the Supporting information to the main text as Table 2. The content of Tables 2 and 3 was adjusted concomitantly. Moreover, the detailed individual data are available in the Data repository resources.
